# The impact of the stress hyperglycemia ratio on mortality and rehospitalization rate in patients with acute decompensated heart failure and diabetes

**DOI:** 10.1186/s12933-023-01908-2

**Published:** 2023-07-26

**Authors:** Qing Zhou, Jie Yang, Wenyao Wang, Chunli Shao, Xinwei Hua, Yi-Da Tang

**Affiliations:** 1 Department of Cardiology, State Key Laboratory of Cardiovascular Disease, National Center for Cardiovascular Diseases, Fuwai Hospital, Graduate School of Peking Union Medical College, Chinese Academy of Medical Sciences and Peking Union Medical College, Beijing, 100037 China; 2 Department of Cardiology and Institute of Vascular Medicine, Peking University Third Hospital, No. 49 Huayuanbei Road, Beijing, 100191 China; 3 State Key Laboratory of Vascular Homeostasis and Remodeling, Peking University, Beijing, 100191 China; 4 NHC Key Laboratory of Cardiovascular Molecular Biology and Regulatory Peptides, Peking University, Beijing, 100191 China; 5 Beijing Key Laboratory of Cardiovascular Receptors Research, Beijing, 100191 China

**Keywords:** Acute decompensated heart failure, Stress hyperglycemia, Mortality, Rehospitalization

## Abstract

**Background:**

The relationship between stress hyperglycemia and long-term prognosis in acute decompensated heart failure (ADHF) patients is unknown. This study investigated the associations of stress hyperglycemia with mortality and rehospitalization rates among ADHF patients with diabetes.

**Methods:**

We consecutively enrolled 1904 ADHF patients. Among them, 780 were with diabetes. Stress hyperglycemia was estimated using the stress hyperglycemia ratio (SHR), which was calculated by the following formula: SHR = admission blood glucose/[(28.7 × HbA1c%) – 46.7]. All diabetic ADHF subjects were divided into quintiles according to the SHR. The primary endpoint was all-cause death at the 3-year follow-up. The secondary endpoints were cardiovascular (CV) death and heart failure (HF) rehospitalization at the 3-year follow-up. A Cox proportional hazards model and restricted cubic spline analysis were used to elucidate the relationship between the SHR and the endpoints in diabetic ADHF patients. Further analyses were performed to examine the relationships between SHR and the outcomes in heart failure with preserved ejection fraction (HFpEF) and heart failure with reduced ejection fraction (HFrEF).

**Results:**

A total of 169 all-cause deaths were recorded during a median follow-up of 3.24 years. Restricted cubic spline analysis suggested a U-shaped association between the SHR and the mortality and rehospitalization rates. Kaplan–Meier survival analysis showed the lowest mortality in the 2nd quintile (*P* = 0.0028). Patients categorized in the highest range (5th quintile) of SHR, compared to those in the 2nd quintile, exhibited the greatest susceptibility to all-cause death (with a hazard ratio [HR] of 2.76 and a 95% confidence interval [CI] of 1.63–4.68), CV death (HR 2.81 [95% CI 1.66–4.75]) and the highest rate of HF rehospitalization (HR 1.54 [95% CI 1.03–2.32]). Similarly, patients in the lowest range (1st quintile) of SHR also exhibited significantly increased risks of all-cause death (HR 2.33, 95% CI 1.35–4.02) and CV death (HR 2.32, 95% CI 1.35–4.00). Further analyses indicated that the U-shape association between the SHR and mortality remained significant in both HFpEF and HFrEF patients.

**Conclusion:**

Both elevated and reduced SHRs indicate an unfavorable long-term prognosis in patients with ADHF and diabetes.

**Supplementary Information:**

The online version contains supplementary material available at 10.1186/s12933-023-01908-2.

## Background

Acute decompensated heart failure (ADHF), is the most common form of acute heart failure (HF), accounting for 50–70% of presentations. It defined as the deterioration of preexisting chronic HF that requires urgent medical attention [1–3]. Stress hyperglycemia, which refers to a temporary elevation in blood glucose levels triggered by physiological or psychological stress, is commonly observed among patients with ADHF [4, 5]. While stress hyperglycemia has been linked to worse outcomes in various acute medical conditions [6–9], its impact on mortality and rehospitalization in ADHF is poorly understood. The stress hyperglycemia ratio (SHR), which evaluates the extent of stress-related hyperglycemia in relation to the severity of illness, has been suggested as a potential indicator for predicting unfavorable outcomes in critically ill individuals [10–12]. However, there are limited data on the relationship between the SHR and mortality and rehospitalization in ADHF. Stress hyperglycemia is found to cause inflammation and endothelial dysfunction, which has been proven to be closely correlated with the prognosis of ADHF [2]. Understanding the impact of the SHR on mortality and rehospitalization in ADHF is important, as it may help clinicians identify high-risk patients and tailor their treatment strategies accordingly. The objective of this study was to investigate the association between the SHR and mortality and rehospitalization in patients with ADHF and. Considering the situations are very different between diabetes and non-diabetes individuals and stress hyperglycemia is more obvious in patients with diabetes, we limited our analytical population to AHDF patients with diabetes.

## Methods

### Study design and participants

**Fig. 1:**
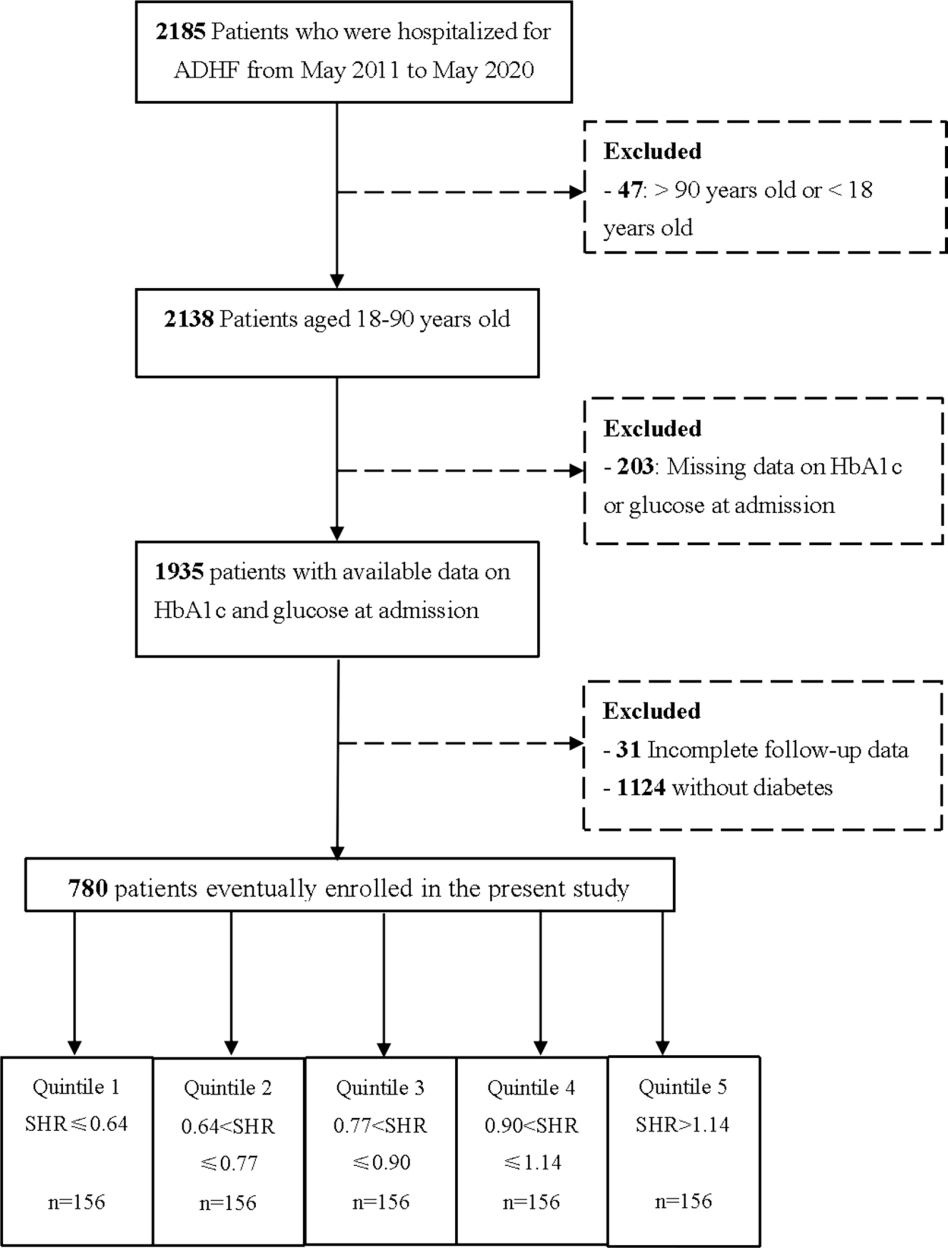
Flowchart of the included population. *ADHF* acute decompensated heart failure, *HbA1c* hemoglobin A1c, *SHR* stress hyperglycemia ratio

This was a prospective observational cohort study conducted at Peking University Third Hospital from May 1, 2011 to May 31, 2020. We consecutively screened 2185 patients who were admitted to the hospital due to ADHF. Participants were assessed for eligibility according to the most up-to-date ESC guidelines available at the time of patient admission. Patients were excluded if they were over 90 or under 18 years old, had missing important laboratory data, had incomplete follow-up data, or without diabetes. These patients were previously diagnosed with chronic heart failure, and their current hospitalizations were due to acute episodes. Details of the population enrolment are provided in Fig. 1. The study was conducted in accordance with the Declaration of Helsinki and was authorized by the Peking University Third Hospital Ethics Review Committee. All participants provided written informed consent.

### Data collection and endpoint definitions

Baseline demographic and clinical data, including age, sex, body mass index (BMI), smoking history, laboratory tests, vital signs, echocardiographic data, comorbidities, and medication history, were collected from an electronic medical recording system by trained physicians. Peripheral venous blood samples were collected after overnight fasting (> 8 h) and were measured in the laboratory department. Diabetes mellitus (DM) is defined as fasting plasma glucose (FPG) ≥ 126 mg/dL, hemoglobin A1c (HbA1c) ≥ 6.5%, or a self-reported history of diabetes. Laboratory tests included admission blood glucose (ABG), HbA1c, N-terminal pro-B-type natriuretic peptide (NT-proBNP), lipid profile, creatinine (Cr), hemoglobin (HGB), troponin T (cTnT), and thyroid functions. We also recorded medication history, including use of insulin, metformin, sodium-glucose cotransporter-2 inhibitors (SGLT2i), other hypoglycemic drugs, angiotensin-converting enzyme inhibitors (ACEI), angiotensin receptor blockers (ARB), angiotensin receptor II blocker—neprilysin inhibitor (ARNI), calcium channel blockers (CCB), aldosterone receptor antagonist (MRA), loop diuretics and thiazide.

### SHR calculation

SHR = admission blood glucose/[(28.7 × HbA1c%) – 46.7]. [10]

### Endpoints and follow-up

Following discharge, we conducted patient follow-ups at 1 and 6 months and annually. This was done through various means, including telephone calls, correspondence, and outpatient visits. The primary endpoint was all-cause death. The secondary endpoints were cardiovascular (CV) death and HF rehospitalization.

### Statistical analyses

The patients were divided into quintiles according to their SHR levels: 1st quintile, SHR ≤ 0.64; 2nd quintile, 0.64 < SHR ≤ 0.77; 3rd quintile, 0.77 < SHR ≤ 0.90; 4th quintile, 0.90 < SHR ≤ 1.14; 5th quintile, SHR > 1.14.

Continuous variables with a normal distribution are presented as the mean ± standard deviation, while nonnormally distributed measurement data are described as the median and interquartile range (IQR). Categorical variables were expressed as quantities and percentages. We used analysis of variance (ANOVA) to assess the differences in continuous variables with a normal distribution across the five groups. For continuous variables that did not have a normal distribution or homogeneity of variance, we performed the rank sum test to explore differences among the groups. The chi-square test was used to evaluate differences in categorical variables.

We performed log-rank tests and Kaplan‒Meier (K-M) survival analyses to explore differences in event-free survival among the different groups. Multivariate Cox proportional hazards models were applied to test the associations of the SHR with the incidence rates of the three primary outcomes. The variables selected in the multivariable Cox proportional hazard models included age, sex, smoking, BMI, NT-proBNP, TG, LDL-C, Cr, FT3, SBP, LVEF, E/E’, coronary heart disease, atrial fibrillation, and use of insulin, ACEI/ARB/ARNI and SGLT2i.

Time to event was defined as days from the date of the blood draw to the endpoint or censored date. Tests for trend were conducted by including the SHR quintiles in the model as an ordinal variable and calculating the Wald statistic. Additionally, we conducted restricted cubic spline (RCS) analyses using five knots placed at the 5th, 27.5th, 50th, 72.5th, and 95th centiles to examine the association between SHR and the endpoints. We also performed multivariate Cox proportional hazards models and RCS analyses to evaluate the impact of the SHR on prognosis in patients with heart failure with preserved ejection fraction (HFpEF) and reduced ejection fraction (HFrEF) as well as mid-range ejection fraction (HFmrEF). HFpEF group contained patients with LVEF ≥ 50%, while the HFrEF / HFmrEF group included those with LVEF < 50% (HFrEF referred to those with LVEF < 40%, and HFmrEF referred to those with LVEF in 40–49%). SPSS Statistics (version 26; SPSS, Chicago, IL) and R (version 4.2.0) were used to perform the statistical analyses. A two-tailed P-value less than 0.05 was used as the threshold for determining statistical significance.

## Results

### Baseline characteristics

A total of 2185 ADHF patients were assessed for eligibility. Ultimately, 780 patients with ADHF and diabetes were included in our main analyses. These patients had a mean age of 68.9 ± 12.7 years. The median duration of follow-up was 3.24 years (interquartile range [IQR]: 2.85 to 3.78 years). Detailed baseline data are shown in Table 1. Among the different quintiles of SHR, the highest levels of NT-proBNP were observed in the 5th quintile (4494 pg/mL [IQR: 1639 to 11,815 pg/mL]), while the lowest levels were found in the 2nd quintile (2116 pg/mL [IQR: 895 to 4755 pg/mL]). Regarding LVEF, the 5th quintile had the lowest mean value at 44.4 ± 15.7%, while the 2nd quintile had the highest mean value at 50.3 ± 17.3%. The proportions of patients with coronary heart disease or myocardial infarction were highest in the 5th quintile of SHR. Additionally, the 1st and 5th quintiles had significantly higher proportions of patients using insulin compared with the other groups.

**Table 1:** Baseline characteristics of the five groups

Characteristics	Overall n = 780	Quintile 1 SHR ≤ 0.64 n = 156	Quintile 2 0.64 < SHR ≤ 0.77 n = 156	Quintile 3 0.77 < SHR ≤ 0.90 n = 156	Quintile 4 0.90 < SHR ≤ 1.14 n = 156	Quintile 5 SHR > 1.14 n = 156	P
SHR	0.84 [0.68, 1.07]	0.57 [0.47, 0.60]	0.72 [0.68, 0.74]	0.84 [0.81, 0.86]	1.01 [0.94, 1.07]	1.40 [1.25, 1.60]	< 0.001
ABG, mmol/L	8.30 [6.55, 11.0]	5.30 [4.57, 6.62]	6.50 [5.80, 7.90]	7.50 [6.70, 8.60]	9.00 [7.90, 10.8]	12.8 [10.5, 16.1]	< 0.001
HbA1C, %	7.64 ± 1.53	8.15 ± 1.81	7.62 ± 1.54	7.53 ± 1.25	7.47 ± 1.41	7.42 ± 1.46	< 0.001
Age, years	68.9 ± 12.7	67.8 ± 13.0	70.2 ± 13.1	67.2 ± 13.1	69.4 ± 11.5	70.0 ± 12.4	0.144
Female	286 (36.7)	61 (39.1)	58 (37.2)	52 (33.3)	61 (39.1)	54 (34.6)	0.764
BMI, kg/m^2^	25.6 ± 4.5	26.0 ± 4.6	25.9 ± 4.5	26.1 ± 4.4	24.9 ± 4.2	25.0 ± 4.5	0.023
Smoking	273 (35.0)	59 (37.8)	53 (34.0)	51 (32.7)	52 (33.3)	58 (37.2)	0.827
Laboratory tests							
NT-proBNP, pg/mL	2820 [1192, 6681]	2584 [1207, 5905]	2116 [895, 4755]	2675 [1195, 5850]	2804 [1165, 7038]	4494 [1639, 11815]	< 0.001
LDL-C, mmol/L	2.10 [1.63, 2.76]	2.03 [1.48, 2.75]	2.02 [1.53, 2.71]	2.27 [1.74, 2.81]	2.13 [1.67, 2.83]	2.09 [1.69, 2.71]	0.209
TC, mmol/L	3.78 ± 1.16	3.78 ± 1.39	3.69 ± 1.07	3.87 ± 1.08	3.79 ± 1.17	3.75 ± 1.09	0.758
HDL-C, mmol/L	0.92 ± 0.26	0.91 ± 0.27	0.93 ± 0.25	0.90 ± 0.23	0.94 ± 0.31	0.91 ± 0.24	0.587
TG, mmol/L	1.26 [0.94, 1.73]	1.19 [0.91, 1.81]	1.17 [0.88, 1.67]	1.36 [1.03, 1.96]	1.30 [1.01, 1.63]	1.22 [0.91, 1.66]	0.049
Cr, μmol/L	108 [88, 157]	112 [87, 164]	105 [89, 147]	103 [85, 144]	104 [86, 148]	118 [97, 201]	0.008
HGB, g/L	121 ± 24	120 ± 24	124 ± 22	124 ± 23	119 ± 22	117 ± 26	0.035
cTnT, ng/mL	0.04 [0.02, 0.15]	0.04 [0.02, 0.11]	0.03 [0.02, 0.07]	0.04 [0.02, 0.13]	0.05 [0.02, 0.14]	0.10 [0.03, 0.62]	< 0.001
TSH, mIU/L	1.90 [1.07, 3.00]	1.90 [1.03, 3.05]	1.93 [1.16, 2.97]	1.92 [1.20, 3.46]	1.91 [1.08, 3.04]	1.54 [0.80, 2.77]	0.233
FT3, pg/mL	2.57 ± 0.58	2.54 ± 0.51	2.63 ± 0.54	2.63 ± 0.50	2.62 ± 0.62	2.46 ± 0.69	0.045
FT4, ng/dL	1.26 [1.12, 1.42]	1.26 [1.12, 1.41]	1.26 [1.12, 1.42]	1.27 [1.12, 1.43]	1.27 [1.15, 1.41]	1.24 [1.12, 1.44]	0.943
Vital signs							
Heart rate, beats/min	86 [75, 108]	90 [77, 102]	90 [79, 102]	83 [74, 108]	86 [77, 102]	85 [69, 116]	0.931
SBP, mmHg	135 ± 21	134 ± 22	134 ± 19	138 ± 19	136 ± 20	135 ± 25	0.484
DBP, mmHg	75 ± 13	73 ± 13	75 ± 14	78 ± 15	75 ± 12	73 ± 12	0.020
Echocardiography							
LVEF	47.5 ± 16.5	48.9 ± 17.7	50.3 ± 17.3	45.5 ± 15.2	48.3 ± 16.2	44.4 ± 15.7	0.008
E/E’	12.7 ± 5.7	12.8 ± 6.0	11.4 ± 4.4	12.4 ± 4.6	12.9 ± 6.4	13.9 ± 6.7	0.005
LVEDD	54.6 ± 8.6	53.8 ± 8.2	53.8 ± 9.1	56.3 ± 8.9	53.8 ± 7.9	55.1 ± 8.6	0.029
Comorbidity							
Hypertension	622 (79.7)	123 (78.8)	114 (73.1)	131 (84.0)	129 (82.7)	125 (80.1)	0.522
Coronary heart disease	556 (71.3)	113 (72.4)	104 (66.7)	111 (71.2)	110 (70.5)	118 (75.6)	0.001
Myocardial infarction	343 (44.0)	65 (41.7)	51 (32.7)	72 (46.2)	66 (42.3)	89 (57.1)	0.003
Atrial fibrillation	236 (30.3)	44 (28.2)	66 (42.3)	45 (28.8)	46 (29.5)	35 (22.4)	0.003
Hyperlipidemia	318 (40.8)	69 (44.2)	55 (35.3)	70 (44.9)	60 (38.5)	64 (41.0)	0.383
Hyperthyroidism	38 (5.05)	3 (2.01)	8 (5.30)	11 (7.33)	7 (4.61)	9 (5.96)	0.304
Chronic kidney disease	160 (20.5)	31 (19.9)	30 (19.2)	23 (14.7)	36 (23.1)	40 (25.6)	0.163
Medication							
Glucose-lowering therapy							
Insulin	356 (45.6)	94 (60.3)	54 (34.6)	52 (33.3)	70 (44.9)	86 (55.1)	< 0.001
Metformin	159 (20.4)	33 (21.2)	30 (19.2)	42 (26.9)	33 (21.2)	21 (13.5)	0.062
SGLT2i	233 (29.9)	42 (26.9)	47 (30.1)	52 (33.3)	50 (32.1)	42 (26.9)	0.636
Other hypoglycemic drugs	264 (33.8)	52 (33.3)	51 (32.7)	60 (38.5)	54 (34.6)	47 (30.1)	0.627
CVD medication							
ACEI/ARB/ARNI	414 (53.1)	85 (54.5)	83 (53.2)	95 (60.9)	78 (50.0)	73 (46.8)	0.135
Beta blocker	447 (57.3)	107 (68.6)	80 (51.3)	86 (55.1)	86 (55.1)	88 (56.4)	0.026
CCB	206 (26.4)	43 (27.6)	39 (25.0)	45 (28.8)	41 (26.3)	38 (24.4)	0.897
MRA	218 (27.9)	42 (26.9)	45 (28.8)	54 (34.6)	35 (22.4)	42 (26.9)	0.197
Loop diuretics	482 (61.8)	94 (60.3)	98 (62.8)	96 (61.5)	100 (64.1)	94 (60.3)	0.946

Data are expressed as mean ± standard deviation, median [interquartile range] or n (%)

*ABG* admission blood glucose, *ACEI* angiotensin-converting enzyme inhibitors, *ARB* angiotensin receptor blockers, *ARNI* angiotensin receptor II blocker—neprilysin inhibitor, *BMI* body mass index, *CCB* calcium channel blockers, *Cr* creatinine, *cTnT* troponin T, *CVD* cardiovascular disease, *DBP* diastolic blood pressure, E/E’ the ratio of early diastolic mitral inflow velocity to septal mitral annulus tissue relaxation velocity in early diastole, *FT3* free triiodothyronine, *FT4* free thyroxine, *HDL-C* high-density lipoprotein cholesterol, *HGB* hemoglobin, *LDL-C* low-density lipoprotein cholesterol, *LVEDD* left ventricular end-diastolic dimension, *LVEF* left ventricle ejection fraction, *MRA* aldosterone receptor antagonist, *NT-proBNP* N-terminal pro-B-type natriuretic peptide, *SBP* systolic blood pressure, *SGLT2i* sodium-glucose cotransporter-2 inhibitors, *SHR* stress hyperglycemia ratio, *TC* total cholesterol, *TG* triglycerides, *TSH* thyroid stimulating hormone

### Primary and secondary outcomes

During the 3-year follow-up period, a total of 169 deaths (21.7% of the cohort) were recorded, with 165 of those deaths (21.2%) attributed to CV causes. Additionally, 231 patients (29.6%) were rehospitalized due to HF.

Among the different SHR quintiles, the highest mortality rate was observed in the 5th quintile, with 46 deaths (29.5% of the group) recorded. The second highest mortality rate was observed in the 1st quintile, with 38 deaths (24.4% of the group). In contrast, the lowest mortality rate was observed in the 2nd quintile, with 20 deaths (12.8% of the group). Similarly, the highest rates of CV mortality and HF rehospitalization were also observed in the 5th quintile. In this group, there were 45 cases of CV deaths (28.8% of the group) and 53 cases of HF rehospitalization (34.0% of the group). Further details regarding deaths and rehospitalizations in the different SHR groups are displayed in Table 2.

**Table 2:** The number of events among the five groups

Endpoints	Overall n = 780	Quintile 1 n = 156	Quintile 2 n = 156	Quintile 3 n = 156	Quintile 4 n = 156	Quintile 5 n = 156	*P* value
All-cause death	169 (21.7)	38 (24.4)	20 (12.8)	30 (19.2)	35 (22.4)	46 (29.5)	0.007
CV death	165 (21.2)	38 (24.4)	20 (12.8)	28 (17.9)	34 (21.8)	45 (28.8)	0.007
HF rehospitalization	231 (29.6)	46 (29.5)	43 (27.6)	40 (25.6)	49 (31.4)	53 (34.0)	0.531

The numbers of events are shown as n (%)

*CV* cardiovascular, *HF* heart failure

**Fig. 2:**
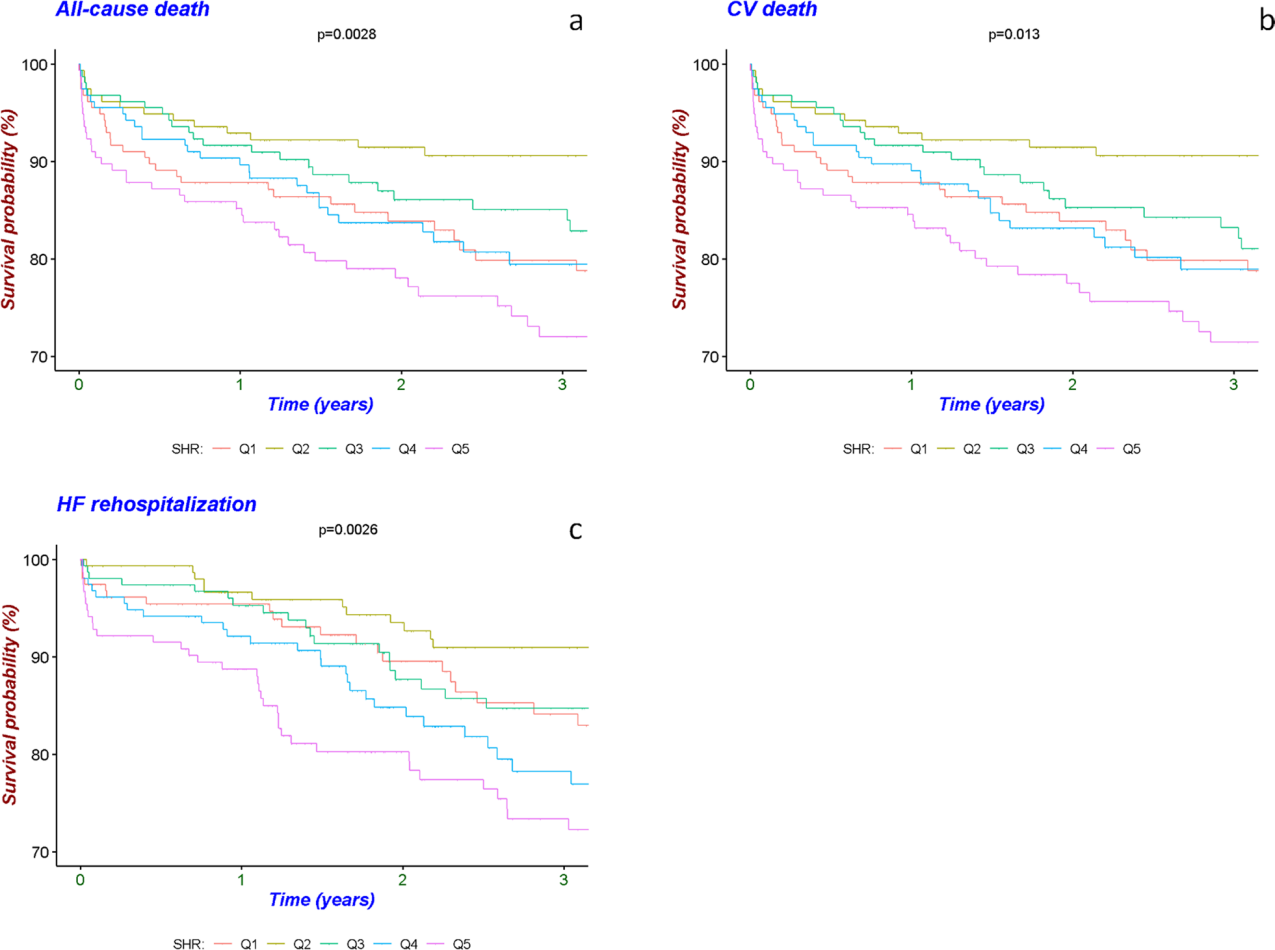
Kaplan‒Meier analyses for different endpoints among the five groups. **a** All-cause death. **b** CV death. **c** HF rehospitalization. Q1–Q5: quintile 1–5

Kaplan–Meier survival analyses were conducted to assess the incidence of all-cause death, CV death, and HF rehospitalization across different quintiles of the SHR. The results indicated a significant difference in the occurrence of these outcomes among the SHR quintiles. Specifically, the 2nd quintile had the lowest incidence rate, while the 5th quintile had the highest incidence rate, with all p-values being less than 0.05. Detailed information regarding these analyses is presented in Fig. 2.

To further analyze the association between SHR quintiles and the outcomes, the Cox proportional hazards model was performed using the 2nd quintile as the reference group. The results demonstrated that individuals in the 1st and 5th quintiles had significantly higher risks of all-cause death (1st quintile: hazard ratio [HR] = 2.33, 95% confidence interval [CI] = 1.35–4.02, p = 0.002; 5th quintile: HR = 2.76, 95% CI = 1.63–4.68, p < 0.001). Similarly, for CV death, patients in the 1st and 5th quintiles also had significantly higher risks (1st quintile: HR = 2.32, 95% CI = 1.35–4.00, p = 0.002; 5th quintile: HR = 2.81, 95% CI = 1.66–4.75, p < 0.001). Regarding HF rehospitalization, the 5th quintile exhibited significantly higher incidences (HR = 1.54, 95% CI = 1.03–2.32, p = 0.036), while no significant differences were found between the 1st quintile and the reference group (HR = 1.35, 95% CI = 0.89–2.05, p = 0.16). Additional information and comprehensive results of the multivariable Cox regression analysis are presented in Table 3.

**Table 3:** Multivariable Cox regression analyses for different endpoints in patients with acute decompensated heart failure and diabetes

Exposure	All-cause death			CV death			HF rehospitalization		
	HR	95% CI	P value	HR	95% CI	P value	HR	95% CI	P value
Q1	2.33	1.35–4.02	0.002	2.32	1.35–4.00	0.002	1.35	0.89–2.05	0.160
Q2	Reference				Reference			Reference	
Q3	1.64	0.92–2.92	0.091	1.75	0.99–3.08	0.054	1.18	0.76–1.82	0.458
Q4	2.13	1.22–3.70	0.007	2.18	1.26–3.78	0.005	1.54	1.02–2.33	0.040
Q5	2.76	1.63–4.68	< 0.001	2.81	1.66–4.75	< 0.001	1.54	1.03–2.32	0.036
P for trend			0.054			0.038			0.109

The results are adjusted for age, sex, smoking, BMI, NT-proBNP, TG, LDL-C, Cr, FT3, SBP, LVEF, E/E’, coronary heart disease, atrial fibrillation, and use of insulin, ACEI/ARB/ARNI and SGLT2i

*CI* confidence interval, *CV* cardiovascular, *HF* heart failure, *HR* hazard ratio, *Q1-Q5*, quintile 1–5

### The SHR as a continuous variable

**Fig. 3:**
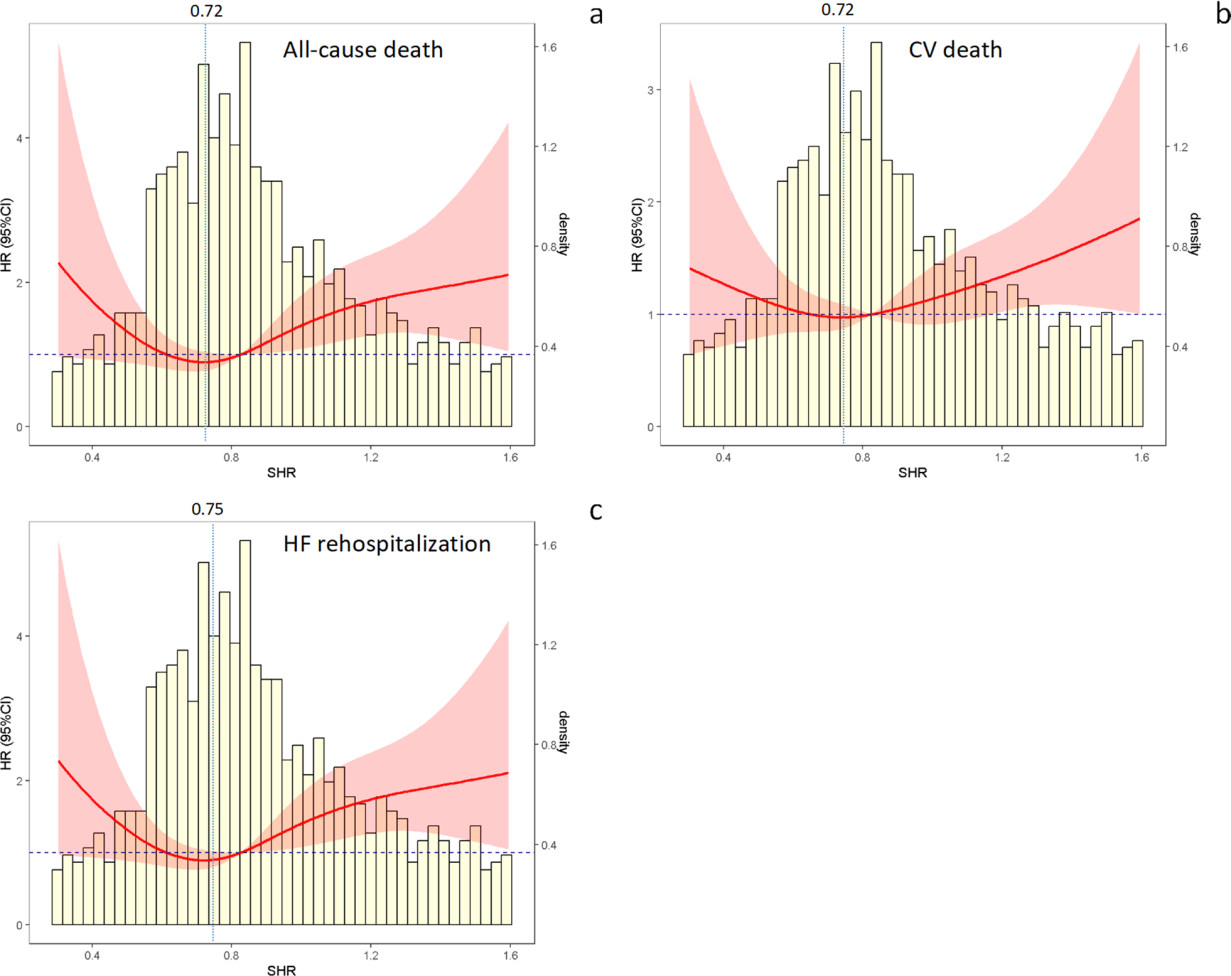
Nonlinear associations of the stress hyperglycemia ratio with different endpoints in the total study population. **a**: All-cause death. **b**: CV death. **c**: HF rehospitalization

In Fig. 3, the results of the RCS analyses indicated that there were U-shaped associations of the SHR with all the outcomes at the 3-year follow-up (all P values for nonlinearity < 0.05). The values of the SHR corresponding to the lowest risks of all-cause death, CV death, and HF rehospitalization on multivariate-adjusted RCS analyses were 0.72, 0.72, and 0.75, respectively.

### The SHR with HFpEF and HFrEF

Further analyses were conducted to investigate the association between SHR and the outcomes in HFpEF and HFrEF/HFmrEF patients separately. The results of multivariate Cox regression models and RCS analyses showed that the U-shaped associations remained significant in both HFpEF and HFrEF/HFmrEF populations. In HFrEF/HFmrEF patients, patients in 1st quintile and 5th quintile had more than three times risk of all-cause death compared to 2nd quintile (1st quintile: hazard ratio [HR] = 3.29, 95% confidence interval [CI] = 1.58–6.84, p = 0.001; 5th quintile: HR = 3.12, 95% CI = 1.51–6.43, p = 0.002). RCS analyses showed a U-shape association between SHR and all three outcomes in HFrEF/HFmrEF patients. In HFpEF patients,patients in 5th quintile also had significantly greater risk of all-cause deaths compared to those in the 2nd quintile (HR = 2.36, 95% CI = 1.05–5.32, p = 0.037). In RCS analyses, U-shape associations were observed in SHR and all-cause death as well as CV death, while J-shape association was observed in HF rehospitalization. Details on the Cox regression and RCS analyses in this part can be found in Additional file 1: Table S1 and Figs. S1, S2.

## Discussion

To our knowledge, this is the first prospective study with a relatively large sample size to investigate the association between SHR and the long-term prognosis of ADHF patients with diabetes. We discovered U-shaped relationships between SHR and the three endpoints in diabetic ADHF patients in this cohort study, implying that both very low and very high SHR were significantly associated with worse long-term prognosis of ADHF. These results indicated that the SHR was an independent predictor of poor long-term prognosis in ADHF patients. More significantly, this research proposed a simple and efficient method for evaluating stress hyperglycemia to optimize risk stratification in ADHF patients with diabetes.

### Associations of SHR with HF

Our study findings are consistent with previous research on SHR in HF patients. We observed that patients with a moderate SHR range (0.64–0.77) had the lowest mortality and rehospitalization rates, which is in line with a recent study published in 2022 [10]. The study revealed that there is a U-shaped relationship between SHR and short-term outcomes in patients with HF. The outcomes included in-hospital cardiac events, acute kidney injury, and systemic infection. They divided 2875 participants into three groups and found that the third tertile had higher risks of in-hospital events. Our research also found a U-shape association between the SHR and long-term outcomes, and the 5th quintile had the highest risk of long-term mortality and rehospitalization. However, there are also some differences between this previous study and our research. First, in the previous study [10], the endpoints were in-hospital events. In our study, the endpoints were all-cause death, CV death and HF rehospitalization at the 3-year follow-up. Second, in the previous study, the researchers utilized logistic regression models and odds ratios to evaluate the association between the SHR and short-term prognosis. In contrast, our study employed Kaplan‒Meier curves, multivariable Cox regression models, and RCS analyses to comprehensively evaluate the long-term effects of the SHR on patient mortality and rehospitalization. Third, this previous study included HF patients without specifically classifying the type of HF. In our study, we restricted our study population to patients with ADHF, which is a subtype of acute HF. With this decision, we aimed to reduce the heterogeneity within the study population and enable more precise conclusions.

In contrast, a study in Spain reported different findings, where the SHR was negatively associated with mortality in acute HF patients and the third tertile had the lowest mortality [13]. This research did not provide specific SHR ranges for each tertile, and the reason why they found that the SHR is negatively associated with HF mortality is that patients with a low SHR suffered from hypoglycemia. The prevalence of type-2 diabetes in subjects with HF is 25–30%, and unsuitable use of hypoglycemic medication often leads to hypoglycemia, which is an important risk factor for cardiovascular events and mortality. In our research, we found that patients in the 1st quintile had a higher mortality risk than those in the 2nd quintile. Therefore, a high SHR suggests the occurrence of stress hyperglycemia, and a low SHR suggests hypoglycemia. Both situations are detrimental to HF patients.

### Associations of SHR with other CVDs

In addition to HF, previous research has also investigated the association between the SHR and long-term prognosis in patients with other CVDs. Yang et al. [14] demonstrated that SHR is associated with the long-term prognosis of acute coronary syndrome (ACS) patients after drug-eluting stent implantation. They reported a U-shaped association between SHR and major cardiovascular and cerebrovascular events (MACCEs) and found that the SHR corresponding to the lowest risk of MACCEs was 0.78. Our study found that the SHR for the lowest risk of mortality was 0.72, partly because Yang et al. [14] excluded patients with glucose at the admission of < 3.0 mmol/L. Another study enrolled 1553 acute myocardial infarction patients [15]. They found that the SHR was a better predictor of in-hospital mortality and morbidity than glycemia at admission, with the third tertile of SHR having the highest incidence of the combined endpoint. Gao et al. [16] consecutively enrolled 1300 patients with ST-segment elevation myocardial infarction (STEMI) treated with percutaneous coronary intervention. The findings of that study indicated that the SHR was closely related to in-hospital major adverse cardiovascular events (MACE) in STEMI patients regardless of diabetic status (diabetic patients: odds ratio = 2.45; 95% CI = 1.24–4.82; p = 0.010; nondiabetic patients: odds ratio = 5.84; 95% CI = 2.50–13.66; p < 0.001) [13]. A recent study revealed that the SHR was independently associated with in-hospital adverse outcomes in patients with acute myocarditis. Compared with an SHR below 1.12, an SHR over 1.12 significantly increased the risk of in-hospital major adverse cardiovascular events (MACE) with an HR of 3.946 [17].

In clinical practice, a relative increase in glucose levels occurs very frequently in hospitalized patients. Cui et al. [12] reported that a high SHR is associated with increased long-term mortality in patients with acute myocardial infarction, which is similar to our conclusion. They divided patients into two groups according to the SHR: a low SHR group and a high SHR group. They found that a high SHR was associated with increased long-term mortality. In our study, we divided patients into five groups based on the SHR: the 1st quintile, 2nd quintile, 3rd quintile, 4th quintile and 5th quintile. We found that both the very low (1st quintile) and very high (5th quintile) SHR groups had a significantly higher risk of all-cause death. In addition, we conducted restricted cubic spline analyses to further demonstrate the U-shaped association between the SHR and mortality. Therefore, in addition to the clinical significance of a high SHR, our study also revealed that a very low SHR, which often indicates hypoglycemia episodes, is also harmful to long-term prognosis. While numerous studies have examined the SHR in other populations, such as patients with acute myocardial infarction, a notable dearth of research specifically exploring the impact of the SHR on ADHF remains. Thus, our study fills an important gap in the literature by shedding light on the unique association between the SHR and outcomes of ADHF.

### Potential mechanisms between SHR and HF

The physiological mechanisms underlying stress hyperglycemia and its association with the long-term prognosis of HF are not fully understood. Stress hyperglycemia is characterized by an acute increase in blood glucose levels that often occurs when patients are under stress and the hypothalamic‒pituitary‒adrenal axis is activated [18]. While it is unclear whether this change directly contributes to myocardial injury, research has shown that acute increases in plasma glucose levels trigger endothelial dysfunction, oxidative stress, and inflammation [19–21], as well as activate coagulation [22–26]. These changes can cause atherosclerosis [27] and cardiomyopathy [28], impair myocardial contractility, facilitate fluid retention, and worsen HF symptoms. These mechanisms may also explain why sodium-glucose cotransporter 2 (SGLT2) inhibitors effectively improve HF symptoms even in nondiabetic patients [29, 30]: SGLT2 inhibitors suppress acute hyperglycemia, decrease oxidative stress [31], and improve cardiac mitochondrial function, leading to left ventricular function improvement [32]. SGLT2 inhibitors ameliorate myocardial injury in nondiabetic myocardial infarction with acute hyperglycemia by suppressing beclin1-dependent autosis [33]. On the other hand, very low SHR is also associated with a worse long-term prognosis in HF patients. Individuals with very low SHR may experience more hypoglycemic episodes. This could be attributed to factors such as incorrect utilization of insulin or oral hypoglycemic medications, extended periods of fasting, or digestive difficulties [34, 35]. This has been demonstrated to increase the risk of death and cardiovascular events in these patients.

**Fig. 4:**
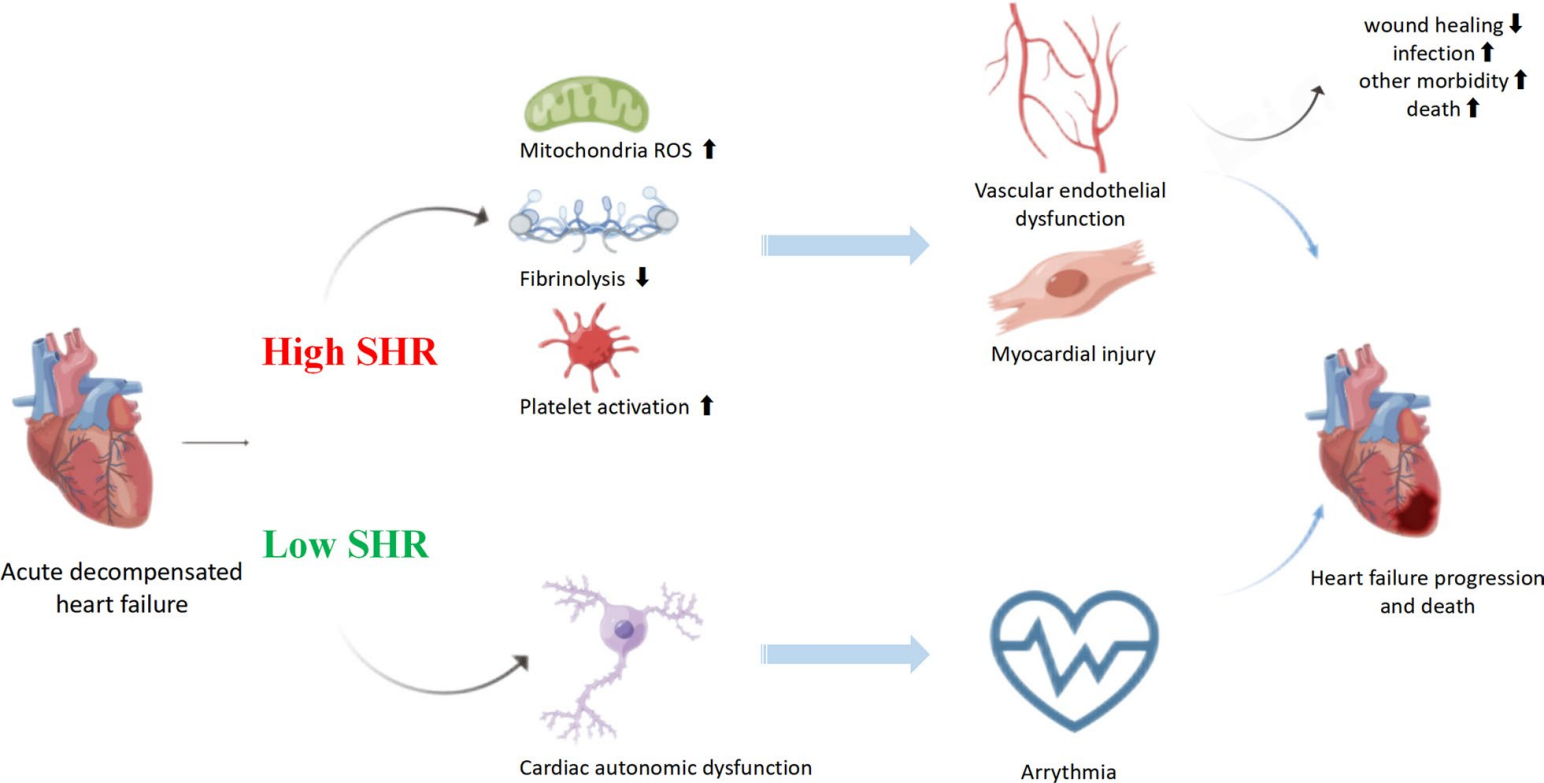
Scientific basis of the association between stress hyperglycemia ratio and all-cause death in patients with acute decompensated heart failure. *SHR* stress hyperglycemia ratio, *ROS* reactive oxygen species

The association between the SHR and all-cause death in patients with ADHF can largely be attributed to the inflammatory response triggered by blood glucose fluctuations[36]. A high SHR indicates stress hyperglycemia, which is often the result of a complex interplay of counterregulatory hormones such as catecholamines, glucocorticoids, and cytokines during illness [37, 38]. The mechanism linking a high SHR to increased mortality can be explained as follows (Fig. 4): (1) Mitochondrial reactive oxygen species (ROS) overproduction: The rapid rise in blood glucose levels causes an overproduction of ROS in the endothelial cells of both large and small blood vessels, as well as in the myocardium. This oxidative stress can lead to endothelial dysfunction, impaired vasodilation, and increased susceptibility to cardiovascular events [39]. (2) Impaired fibrinolysis: Stress hyperglycemia often results in impaired fibrinolysis, the process responsible for breaking down blood clots. Hypofibrinolysis and prolonged clot lysis time are well-established features of type 2 diabetes [22]. Studies have shown that individuals with type 2 diabetes exhibit abnormally high levels of plasminogen activator inhibitor-1 (PAI-1), indicating a state of hypofibrinolysis [40]. Treatment of hyperglycemia with medications such as glipizide or metformin reduces PAI-1 levels, suggesting that glucose-lowering medications play a role in improving fibrinolysis [41]. (3) Platelet activation: Hyperglycemia may act as a causative factor for platelet activation in vivo. It contributes to nonenzymatic glycation of platelet glycoproteins. This process alters the structure, conformation, and membrane lipid dynamics of platelets, leading to their activation [42]. (4) Endothelial dysfunction, impaired fibrinolysis, and platelet activation are independent factors contributing to atherothrombotic events, leading to the deterioration of cardiac function and ultimately increasing the risk of mortality [36, 43]. Additionally, uncontrolled blood glucose levels can have detrimental effects on wound healing, increase the risk of infections, and exacerbate other comorbidities, which can contribute to noncardiovascular-related deaths [44].

Alternatively, a low SHR indicates the occurrence of hypoglycemic episodes due to rigorous blood glucose control. Individuals with diabetic autonomic dysfunction are at a higher risk of experiencing severe hypoglycemia due to a lack of awareness of hypoglycemia [36]. Patients with cardiac autonomic dysfunction are more prone to developing arrhythmias in response to hypoglycemia. Additionally, individuals with a history of diabetes and existing endothelial dysfunction, which impairs blood vessel function, may experience more severe hypoglycemic responses and have an increased susceptibility to ischemic events compared to those without a history of diabetes [45]. Arrhythmias, as well as ischemic events, often lead to deterioration of cardiac function and death.

In summary, the association between the SHR and all-cause mortality in ADHF patients can be attributed to the inflammatory response triggered by blood glucose fluctuations. Mechanisms such as ROS production, impaired fibrinolysis, platelet activation, autonomic dysfunction and noncardiovascular complications contribute to overall mortality.

The present study suggests that the inflection point for the lowest mortality in HF patients is an SHR of 0.72, with the ideal range for SHR being 0.64 to 0.77. An SHR of greater than 0.77 may indicate the occurrence of stress hyperglycemia, while an SHR of less than 0.64 may indicate poor glycemic control. An SHR between 0.64 and 0.77 suggests that the patient's glycemic control is currently appropriate. Calculating the SHR in ADHF patients may help clinicians to adjust hypoglycemic treatment and achieve better glycemic control. Additionally, the SHR is a good prognostic marker and can contribute to risk stratification in ADHF patients. We found that patients with a high SHR often have more comorbidities, smaller LVEF, and less use of anti-HF drugs. Using the SHR as a marker can help clinicians provide appropriate treatment and nursing care levels to reduce mortality rates and save on medication costs.

### Study limitations

There are several limitations to our study that need to be considered. First, since this was an observational study, we could not establish a causal relationship between the SHR and the long-term prognosis of ADHF patients. Further studies using experimental designs are needed to confirm our findings. Besides, the current study measured ABG and HbA1c and calculated the SHR only once at baseline, which may not accurately reflect the changes in glycemic control over time. Future studies should consider multiple measurements of the SHR and glycemic control over time to better understand this association.

Furthermore, although we made considerable efforts to minimize confounding through several strategies, importantly, there are potential confounding factors that were not measured or considered in our study. For example, socioeconomic status, including variations in income, education level, and access to healthcare resources, can influence patient outcomes and treatment decisions. In addition, health behaviors such as alcohol consumption, diet, and physical activity levels can impact the progression of heart failure. For medication use, although we adjusted for common medications for cardiovascular diseases and diabetes in the multivariable Cox regression models, some medications that are not commonly used were not analyzed. Additionally, differences in clinical practices and treatment protocols among healthcare providers may introduce confounding effects. Also, genetic variations and individual genetic profiles can contribute to differences in treatment responses. Therefore, there is a need for caution in interpreting the findings of our study. We call for further research to explore and identify additional confounding factors that may be relevant for ADHF patients.

## Conclusions

In summary, our study found U-shaped associations between the SHR and all-cause death, CV death, and HF rehospitalization in ADHF patients with diabetes. Our findings suggest that monitoring SHR could aid clinicians in assessing the risk of adverse outcomes and adjusting hypoglycemic treatment to improve long-term prognosis in diabetic ADHF patients.

## Supplementary Information

**Additional file 1: Table S1.** Multivariable Cox regression analyses in diabetic patients with HFrEF/HFmrEF and HFpEF. **Figure S1.** Restricted cubic spline analyses in diabetic patients with HFrEF/HFmrEF. **Figure S2.** Restricted cubic spline analyses in diabetic patients with HFpEF.

## Data Availability

The datasets used and/or analyzed in the study are available from the corresponding author upon reasonable requests.

## Abbreviations

ABG: Admission blood glucose
ACEI: Angiotensin-converting enzyme inhibitors
ADHF: Acute decompensated heart failure
ARB: Angiotensin receptor blockers
ARNI: Angiotensin receptor II blocker—neprilysin inhibitor
BMI: Body mass index
CCB: Calcium channel blockers
Cr: Creatinine
cTnT: Troponin T
CV: Cardiovascular
DBP: Diastolic blood pressure
E/E’: The ratio of early diastolic mitral inflow velocity to septal mitral annulus tissue relaxation velocity in early diastole
FT3: Free triiodothyronine
FT4: Free thyroxine
HDL-C: High-density lipoprotein cholesterol
HF: Heart failure
HGB: Hemoglobin
LDL-C: Low-density lipoprotein cholesterol
LVEDD: Left ventricular end-diastolic dimension
LVEF: Left ventricle ejection fraction
MRA: Aldosterone receptor antagonist
NT-proBNP: N-terminal pro-B-type natriuretic peptide
PAI-1: Plasminogen activator inhibitor-1
SBP: Systolic blood pressure
SGLT2i: Sodium-glucose cotransporter-2 inhibitors
SHR: Stress hyperglycemia ratio
TC: Total cholesterol
TG: Triglycerides
TSH: Thyroid stimulating hormone
