# Supplementary material for: The impact of the stress hyperglycemia ratio on mortality and rehospitalization rate in patients with acute decompensated heart failure and diabetes
Source: Cardiovasc Diabetol. 2023 Jul 26;22:189. doi: 10.1186/s12933-023-01908-2 (PMC10373236; doi:10.1186/s12933-023-01908-2)
Supplement: Supplementary file 1 — Additional file 1: Table S1. Multivariable Cox regression analyses in diabetic patients with HFrEF/HFmrEF and HFpEF. Figure S1. Restricted cubic spline analyses in diabetic patients with HFrEF/HFmrEF. Figure S2. Restricted cubic spline analyses in diabetic patients with HFpEF. [file 12933_2023_1908_MOESM1_ESM.docx]

**Table S1.** Multivariable Cox regression analyses in diabetic patients with HFrEF/HFmrEF and HFpEF.

|  | All-cause death | | | CV death | | | HF rehospitalization | | |
| --- | --- | --- | --- | --- | --- | --- | --- | --- | --- |
| Exposure | HR | 95% CI | P value | HR | 95% CI | P value | HR | 95% CI | P value |
| **HFrEF/HFmrEF** |  |  |  |  |  |  |  |  |  |
| Q1 | 3.29 | 1.58-6.84 | 0.001 | 3.29 | 1.58-6.85 | 0.001 | 1.95 | 1.13-3.38 | 0.016 |
| Q2 | Reference |  |  | Reference |  |  |  | Reference |  |
| Q3 | 2.42 | 1.12-5.22 | 0.024 | 2.68 | 1.26-5.71 | 0.010 | 1.22 | 0.66-2.25 | 0.518 |
| Q4 | 2.75 | 1.31-5.78 | 0.007 | 2.75 | 1.31-5.79 | 0.008 | 1.79 | 1.03-3.11 | 0.040 |
| Q5 | 3.12 | 1.51-6.43 | 0.002 | 3.13 | 1.52-6.45 | 0.002 | 1.68 | 0.98-2.88 | 0.059 |
| P for trend |  |  | 0.271 |  |  | 0.272 |  |  | 0.634 |
| **HFpEF** |  |  |  |  |  |  |  |  |  |
| Q1 | 1.69 | 0.74-3.87 | 0.211 | 1.70 | 0.74-3.89 | 0.208 | 0.59 | 0.30-1.15 | 0.122 |
| Q2 | Reference |  |  | Reference |  |  |  | Reference |  |
| Q3 | 0.79 | 0.29-2.11 | 0.631 | 0.90 | 0.35-2.32 | 0.819 | 0.78 | 0.40-1.51 | 0.459 |
| Q4 | 1.27 | 0.53-3.07 | 0.594 | 1.27 | 0.53-3.07 | 0.594 | 0.85 | 0.45-1.60 | 0.614 |
| Q5 | 2.36 | 1.05-5.32 | 0.037 | 2.49 | 1.12-5.57 | 0.025 | 1.77 | 0.95-3.31 | 0.071 |
| P for trend |  |  | 0.105 |  |  | 0.076 |  |  | 0.093 |

*CI* confidence interval, *CV* cardiovascular, *HF* heart failure, *HFrEF* heart failure with reduced ejection fraction, *HFmrEF* heart failure with mid-range ejection fraction, *HFpEF* heart failure with preserved ejection fraction, *HR* hazard ratio, *Q1-Q5*, quintile 1-5.

The results are adjusted for age, sex, smoking, BMI, NT-proBNP, TG, LDL-C, Cr, FT3, SBP, LVEF, E/E’, coronary heart disease, atrial fibrillation, and use of insulin, ACEI/ARB/ARNI and SGLT2i.


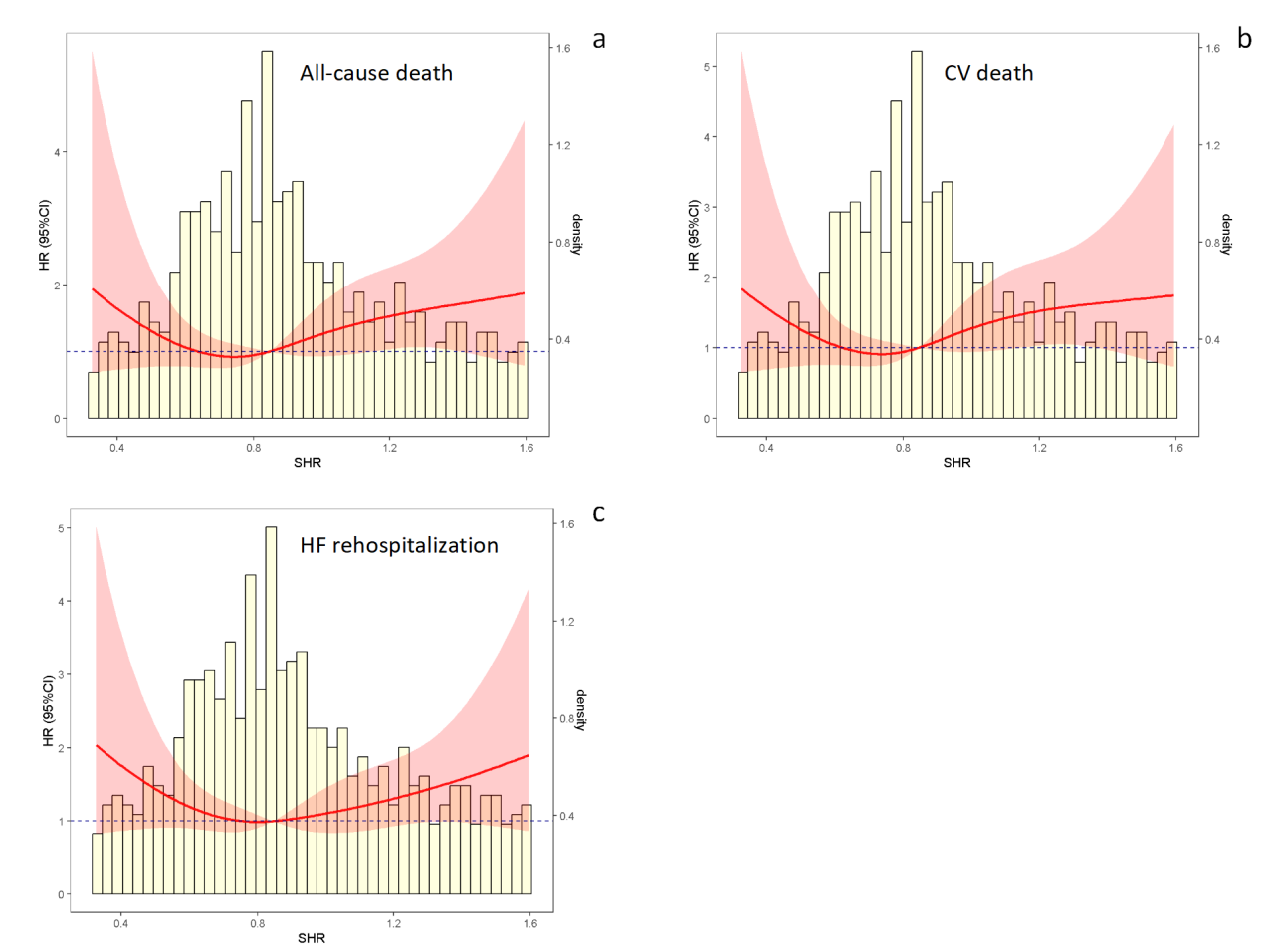


**Figure S1.** Restricted cubic spline analyses in diabetic patients with HFrEF/HFmrEF.

*CI* confidence interval, *CV* cardiovascular, *HF* heart failure, *HFrEF* heart failure with reduced ejection fraction, *HFmrEF* heart failure with mid-range ejection fraction, *HR* hazard ratio, *SHR* stress hyperglycemia ratio.


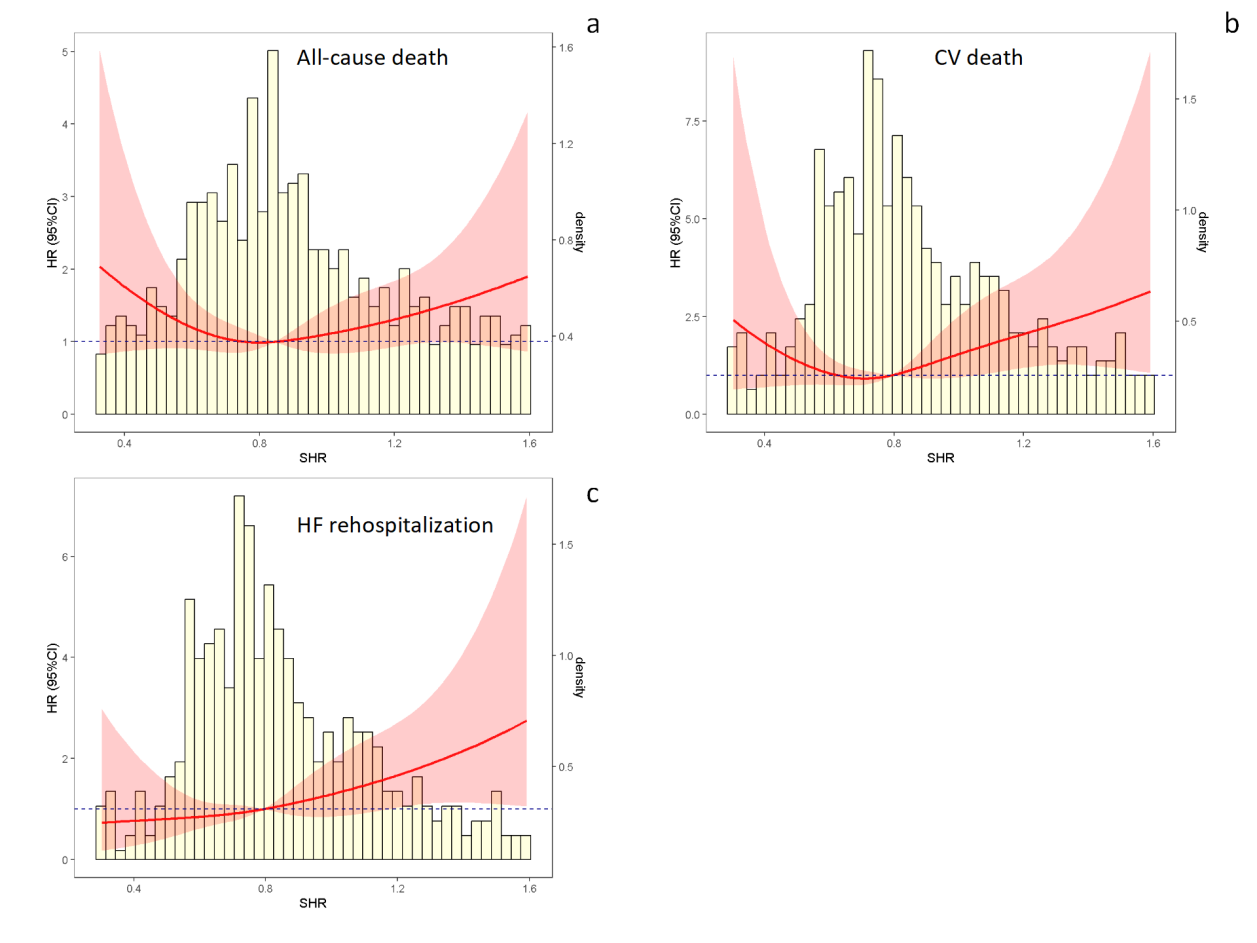


**Figure S2.** Restricted cubic spline analyses in diabetic patients with HFpEF.

*CI* confidence interval, *CV* cardiovascular, *HF* heart failure, *HFpEF* heart failure with preserved ejection fraction, *HR* hazard ratio, *SHR* stress hyperglycemia ratio.
